# Novel 4-thiazolidinone derivatives as agonists of benzodiazepine receptors: Design, synthesis and pharmacological evaluation

**DOI:** 10.17179/excli2016-692

**Published:** 2017-01-13

**Authors:** Mehrdad Faizi, Reza Jahani, Seyed Abbas Ebadi, Sayyed Abbas Tabatabai, Elham Rezaee, Mehrnaz Lotfaliei, Mohsen Amini, Ali Almasirad

**Affiliations:** 1 Department of Pharmacology and Toxicology, School of Pharmacy, Shahid Beheshti University of Medical Sciences, Tehran, Iran; 2 Department of Medicinal Chemistry, Faculty of Pharmacy, Pharmaceutical Sciences Branch, Islamic Azad University, Tehran, Iran; 3 Department of Pharmaceutical Chemistry, School of Pharmacy, Shahid Beheshti University of Medical Sciences, Tehran, Iran; 4 Department of Medicinal Chemistry, Faculty of Pharmacy, Tehran University of Medical Sciences, Tehran, Iran

**Keywords:** benzodiazepine, sedative-hypnotic, anticonvulsant, 4-thiazolidinone derivatives, synthesis

## Abstract

A new series of 4-chloro-N-(2-(substitutedphenyl)-4-oxothiazolidin-3-yl)-2-phenoxybenzamide derivatives were designed, synthesized and biologically evaluated as anticonvulsant agents. The designed compounds have the main essential functional groups for binding to the benzodiazepine receptors and 4-thiazolidinone ring as an anticonvulsant pharmacophore. Some of the new synthesized compounds showed considerable anticonvulsant activity in electroshock and pentylenetetrazole-induced lethal convulsion tests. Compound **5i**, 4-chloro-N-(2-(4-methoxyphenyl)-4-oxothiazolidin-3-yl)-2-phenoxybenzamide, with the best activity was selected for evaluation of other benzodiazepine pharmacological effects. This compound induced significant sedative-hypnotic activity. However, it does not impair the learning and memory in the experimental condition. Flumazenil was able to antagonize the sedative-hypnotic and anticonvulsant effects of compound **5i** indicating that benzodiazepine receptors are highly involved in the pharmacological properties of the novel compounds.

## Introduction

Finding of novel anticonvulsant agents and new ligands for the benzodiazepine (BZD) binding site of the GABA receptor has been an interesting field of drug design in the recent decade. These compounds have important pharmacological effects for the treatment of numerous central nervous system (CNS) disorders such as anxiety, insomnia and epilepsy (Smith et al., 1995[19]; Rudolph and Knoflach, 2011[16]). However, BZD's usefulness is limited by a broad range of unwanted side effects; including sedation, amnesia, ataxia and risk of drug dependence (Alerno et. al., 2012[2]). Therefore, it is important to synthesize novel ligands of the BZD receptor with antiepileptic activity and less undesirable effects.

Classical BZDs are considered as positive allosteric modulators by binding to a distinct site on the GABA_A_ receptors (Guerrini et. al., 2006[10]), which are the greatest population of brain inhibitory neurotransmitter receptors (Guerrini et. al., 2007[9]). The GABA_A_ receptors are symmetric heteropentamers that are made of different subunits “(α1-6, β1-3, γ1-3, δ, ε, θ, π and ρ1-3) with an integral channel that is permeable to chloride ions” (Rudolph and Knoflach, 2011[16]). Most of the GABA_A _receptors are made of α, β and γ subunits, and are arranged around the selective chloride ion gate with the stoichiometry of 2 α subunits, 2 β subunits and 1 γ subunit. The binding site of BZDs is located between α and γ subunits (Rudolph and Knoflach, 2011[16]; Alerno et. al., 2012[2]; Grunwald et al., 2006[8]).

**Figure 1:**
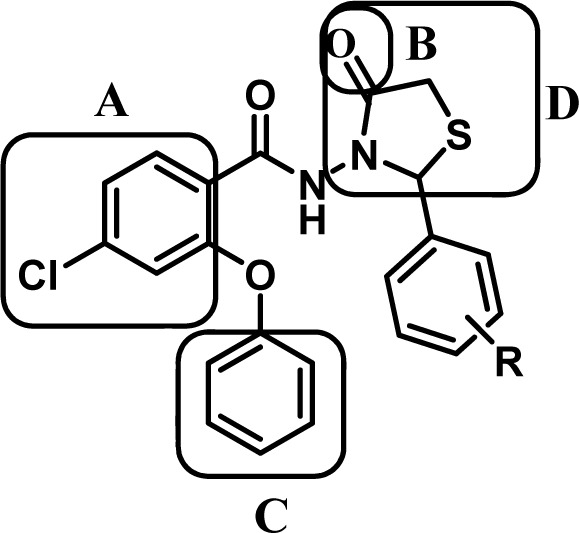
The structure of the novel compounds consist of A: an aromatic ring, B: a coplanar proton accepting group and C: a second out-of-plane aromatic ring, D: adjunct thiazolidinone pharmacophore

There are several pharmacophore models proposed for the binding of ligands to benzodiazepine receptors. Two of these models are more commonly seen in high affinity benzodiazepine ligands. The first one is an aromatic ring with a suitable distance (5Å) from a proton accepting functional group located in the same plane and the second one is presence of the aromatic ring located in a plane perpendicular to the plane of the first aromatic ring (Akbarzadeh et al., 2003[1]). Furthermore, the previously reported article revealed that thiazolidinone derivatives exhibit anticonvulsant activity (Tripathi et al., 2014[21]). Based on these evidences and our earlier published papers on flexible novel heterocyclic compounds (Akbarzadeh et al., 2003[1]; Almasirad et al., 2004[4]; Zarghi et al., 2005[24]; Almasirad et al., 2007[5]; Zarghi et al., 2008[22]; Mahdavi et al., 2010[13]; Faizi et al., 2012[7]; Tabatabai et al., 2013[20]; Zarghi et al., 2008[22][23]; Faizi, et al., 2015[6]) , some new thiazolidinone derivatives that have the essential pharmacophore groups for binding to the BZD's receptors were developed (Figure 1(Fig. 1)). Design of the novel compounds was based on hybridation between 4-thiazolidinone and 2-phenoxyphenyl pharmacophores. Since both pharmacophores have shown anticonvulsant activity, it is expected that the final structures demonstrate comprehensive anticonvulsant effects. Further, the BBB permeably and physicochemical properties of compounds for prediction of oral bioavailability were evaluated. In order to determine the anticonvulsant effects of synthesized compounds, the penthylentetrazole (PTZ)-induced lethal convulsion and maximal electroshock (MES) tests were performed and the results were compared with diazepam, a well-known BZD agonist. The most potent compound was selected to evaluate its effect on learning and memory and the hypnotic activity by passive avoidance test and potentiating of the pentobarbital sleeping time model respectively. Also, the neurotoxicity of this compound was evaluated by the rotarod test and the involvement of BZD receptor in the effects of the novel compounds was confirmed by using flumazenil as an antagonist of BZD receptor.

## Materials and Methods

### Chemistry

Melting points were taken on an Electrothermal 9300 apparatus (Ontario, Canada) and are uncorrected. Infrared spectra and elemental analysis were obtained using Shimadzu FT-IR 8400S spectrographs (KBr disks) and elemental analyser (Costech, Italy) respectively. ^1^H-NMR spectra was recorded on a Bruker FT-500 MHz instrument (Bruker Biosciences, USA) using chloroform-d as solvents. Mass were obtained using a 5973 network mass selective detector at 70 eV (Agilent Technology). All chemicals and reagents were obtained commercially from Sigma/Aldrich or Merck Company and were used without further purification. All of the intermediates were synthesized according to our previously reported articles (Akbarzadeh et al., 2003[1]; Almasirad et al., 2006[3]).

#### General Procedures for the Synthesis of 5a-5j

A mixture of hydrazide **4** (3.80 mmol) and corresponding aldehyde (3.80 mmol) in absolute ethanol was stirred at room temperature for 2 to 20 hours in the presence of hydrochloric acid (2 drops) as a catalyst. After the end of the reaction the mixture was concentrated, neutralized by a 10 % aqueous (aq) solution of sodium bicarbonate and the resulting hydrazone was washed with water, dried and used in the next step without further purification. To a suspension of corresponding hydrazone (2.8 mmol) and Thioglycolic acid (0.38 ml, 5.60 mmol) in dry toluene, catalytic amounts of anhydrous ZnCl_2_ was added and the mixture was refluxed in a round-bottomed flask equipped with a Dean-Stark apparatus for 20 hours. After this time, the solvent was evaporated and NaHCO_3_ 10 % (aq) was added. Finally the precipitate was filtered and recrystallized from ethyl acetate.

#### 4-Chloro-N-(4-oxo-2-phenylthiazolidin-3-yl)-2-phenoxybenzamide (5a)

Yield 64 %; m. p. 102-104° C; **FT-IR** (KBr) (ν, cm^-1^): 3311 (NH), 1726 (C=O), 1652 (C=O); **^1^H-NMR **(CDCl_3_, 500MHz): δppm 9.05 (s, 1H, NH), 8.14 (d, *J* = 10.5 Hz, 1H, aromatic), 7.38-7.27 (m, 4H, aromatic), 7.24-7.21 (m, 4H, aromatic), 7.14 (dd, *J* = 8.5, 2.0 Hz, 1H, aromatic), 6.76 (dd, *J* = 9.0, 3.5 Hz, 2H, aromatic), 6.64 (d, *J* = 2.0 Hz, 1H, aromatic), 6.03 (s, 1H, Ar-CH) , 3.92, 3.75 (2d, *J* = 14.5 Hz, 2H, SCH_2_); **MS**: m/z (%) 426 (M^+^+2,11), 424 (M^+^,33), 247(35), 233(80), 231(100), 196(43), 168(62), 155(37), 139(67), 121(29), 93(90), 77(64). Anal. Calcd for C_22_H_17_ClN_2_O_3_S: C, 62.19; H, 4.03; N, 6.59. Found: C, 62.40; H, 3.96; N, 6.45.

#### 4-Chloro-N-(2-(4-hydroxyphenyl)-4-oxothiazolidin-3-yl)-2-phenoxybenzamide (5b)

Yield 50 %; m. p. 126-128° C; **IR** (KBr) (ν, cm^-1^): 3320, (OH, NH), 1728 (C=O), 1661 (C=O); **^1^H NMR** (CDCl_3_, 500MHz): δppm 9.00 (s,1H, NH), 8.14 (d, *J* = 8.5 Hz, 1H, aromatic), 7.37 (m, 2H, aromatic), 7.30-7.16 (m, 3H, aromatic), 7.14 (dd, *J* = 8.5,2.0 Hz, 1H, aromatic), 6.78 (d, *J* = 7.5 Hz, 2H, aromatic), 6.75 (d, *J* = 7.8 Hz, 2H, aromatic) 6.74 (d, *J* = 2.0 Hz, 1H, aromatic), 6.02 (s, 1H, Ar-CH), 3.90, 3.73 (2d, *J* = 16.0 Hz, 2H, SCH_2_), 3.71 (s, 1H, OH); **MS**: m/z (%) 442 (M^+^+2, 6), 440 (M^+^,18), 33 (100), 231 (33), 208 (22), 168 (28), 155 (27), 139 (40), 111 (32), 97 (55), 84 (71), 69 (91), 57 (99). Anal. Calcd for C_22_H_17_ClN_2_O_4_S: C, 59.93; H, 3.89; N, 6.35. Found: C, 59.71; H, 3.93; N, 6.41.

#### N-(2-(3,5-Di-tert-butyl-4-hydroxyphenyl)-4-oxothiazolidin-3-yl)-4-chloro-2 phenoxybenzamide (5c)

Yield 50 %; m. p. 188-200° C; **IR** (KBr) (ν, cm^-1^): 3502, (OH, bonded), 3370 (OH, non-bonded and NH), 1732 (C=O), 1658 (C=O); **^1^H-NMR** (CDCl_3_, 500MHz): δppm 8.99 (s, 1H, NH), 8.19 (d, *J* = 8.5 Hz, 1H, aromatic), 7.36 (t, 2H, aromatic), 7.24 (t, 1H, aromatic), 7.17 (s, 2H, aromatic), 7.13 (dd, *J* = 8.5, 2.0 Hz, 1H, aromatic), 6.78 (d, *J* = 8.0 Hz, 2H, aromatic), 6.58 (d, *J* = 2.0 Hz, 1H, aromatic), 5.99 (s, 1H, Ar-CH), 5.28 (s, 1H, OH), 3.90, 3.70 (2d, *J* = 16.0 Hz, 2H, SCH_2_), 1.28 (s, 18H, CH_3_); **MS**: m/z (%) 554 (M^+^+2,4), 552 (M^+^,12), 306 (100), 290 (62), 263 (24), 248 (87), 233 (77), 231 (99), 216 (16), 196 (20), 168 (28), 139 (31), 93 (27), 77 (16), 57 (64). Anal. Calcd for C_30_H_33_ClN_2_O_4_S: C, 65.14; H, 6.01; N, 5.06. Found: C, 65.50; H, 5.96; N, 5.00.

#### 4-Chloro-N-(2-(4-(dimethylamino)phenyl)-4-oxothiazolidin-3-yl)-2-phenoxybenzamide (5d)

Yield 50 %; m. p. 152-153° C; **IR** (KBr) (ν, cm^-1^): 3381 (NH), 1717 (C=O), 1667 (C=O); **^1^H-NMR** (CDCl_3_, 500MHz): δppm 9.01 (s, 1H, NH), 8.14 (d, *J* = 8.5 Hz, 1H, aromatic), 7.32 (m, 2H, aromatic), 7.24-7.14 (m, 3H, aromatic), 7.12 (dd, *J* = 8.5, 2.0 Hz, 1H, aromatic), 6.77 (dd, *J* = 6.5, 2.0 Hz, 2H, aromatic), 6.61(d, *J* = 1.5 Hz, 1H, aromatic) 6.49 (d, *J* = 6.5Hz, 2H, aromatic), 5.97 (s, 1H, Ar-CH), 3.92, 3.71 (2d, *J* = 16.0 Hz, 2H, SCH_2_), 2.89 (s, 6H, CH_3_); **MS**: m/z (%) 469 (M^+^+2, 3), 467 (M^+^, 9), 336 (16), 233 (100), 231 (99), 196 (32), 168 (54), 147 (79), 134 (28), 93 (38), 77 (41), 57 (34). Anal. Calcd for C_24_H_22_ClN_3_O_3_S: C, 61.60; H, 4.74; N, 8.98. Found: C, 61.25; H, 4.80; N, 8.93.

#### N-(2-(2-bromophenyl)-4-oxothiazolidin-3-yl)-4-chloro-2-phenoxybenzamide (5e)

Yield 50 %; m. p. 125-127° C; **IR** (KBr) (ν, cm^-1^): 3555 (NH), 1731 (C=O), 1672 (C=O); **^1^H-NMR** (CDCl_3_, 500MHz): δppm 9.14 (s, 1H, NH), 8.15 (d, *J* = 8.5 Hz, 1H, aromatic), 7.44(d, *J* = 7.5 Hz, 1H, aromatic), 7.42-7.20 (m, 4H, aromatic), 7.19-7.02 (m, 3H, aromatic), 6.87 (d, *J* = 8.5 Hz, 2H, aromatic), 6.68 (d, *J* = 2.0 Hz, 1H, aromatic) 6.47 (s, 1H, Ar-CH), 3.89, 3.75 (2d, *J* = 16.0 Hz, 2H, SCH_2_); **MS**: m/z (%) 506 (M^+^+4, 7), 504(M^+^+2, 28), 502(M+, 21), 233 (38), 231 (100), 168 (19), 139 (28), 121 (16), 93 (33), 77 (21), 57 (18). Anal. Calcd for C_22_H_16_BrClN_2_O_3_S: C, 52.45; H, 3.20; N, 5.56. Found: C, 52.61; H, 3.16; N, 5.52.

#### 4-Chloro-N-(2-(4-chlorophenyl)-4-oxothiazolidin-3-yl)-2-phenoxybenzamide (5f)

Yield 50 %; m. p. 113-115° C;** IR** (KBr) (ν, cm^-1^): 3284 (NH), 1722 (C=O), 1672 (C=O); **^1^H-NMR** (CDCl_3_, 500MHz): δppm 9.03 (s, 1H, NH), 8.13 (d, *J* = 8.5Hz, 1H, aromatic), 7.41-7.15(m, 8H, aromatic), 6.77 (d, *J* = 8.0 Hz, 2H, aromatic), 6.68 (d, *J* = 2.0 Hz, 1H, aromatic), 6.01 (s, 1H, Ar-CH), 3.91, 3.73 (2d, *J* = 16.0 Hz, 2H, SCH_2_); **MS**: m/z (%) 462 (M^+^+4,4), 460 (M^+^+2, 24), 458 (M^+^,36), 247 (44), 233 (88), 231 (100), 212 (61), 196 (24), 168 (33), 155 (30), 139 (51), 93 (99), 77 (31). Anal. Calcd for C_22_H_16_Cl_2_N_2_O_3_S: C, 57.52; H, 3.51; N, 6.10. Found: C, 57.61; H, 3.43; N, 5.91.

#### 4-Chloro-N-(2-(3-nitrophenyl)-4-oxothiazolidin-3-yl)-2-phenoxybenzamide (5g)

Yield 50 %; m. p. 99-100° C;** IR** (KBr) (ν, cm^-1^): 3321 (NH), 1726 (C=O), 1665 (C=O), 1531, 1352 (NO_2_); 1**H-NMR **(CDCl_3_, 500MHz): δppm 9.12 (s, 1H, NH), 8.21 (s, 1H, aromatic), 8.08(d, *J* = 8.5Hz , 1H, aromatic), 8.01 (d, *J* = 8.5 Hz, 1H, aromatic), 7.72 (d, *J* = 8.5 Hz, 1H, aromatic), 7.40-7.21 (m, 4H, aromatic), 7.15 (d, *J* = 7.5 Hz, 1H, aromatic), 6.73 (d, *J* = 7.5 Hz, 2H, aromatic), 6.74 (s, 1H, aromatic), 6.11 (s, 1H, Ar-CH), 3.91, 3.80 (2d, *J *= 16.0 Hz, 2H, SCH_2_). **MS**: m/z (%) 471 (M^+^+2, 5), 469 (M^+^, 15), 247 (15), 233 (71), 231 (100), 196 (17), 168 (38), 139 (32), 93 (87), 70 (70). Anal. Calcd for C_22_H_16_Cl_1_N_3_O_5_S: C, 56.23; H, 3.43; N, 8.94. Found: C, 57.17; H, 3.23; N, 8.85.

### 4-Chloro-N-(2-(4-nitrophenyl)-4-oxothiazolidin-3-yl)-2-phenoxybenzamide (5h)

Yield 50 %; m. p. 168-170° C; **IR** (KBr) (ν, cm^-1^): 3398 (NH), 1731 (C=O), 1679 (C=O), 1528,1349 (NO_2_); **^1^H-NMR **(CDCl_3_, 500MHz): δppm 9.04 (s,1H, NH), 8.11(d, *J* = 9.0 Hz , 1H, aromatic), 8.03 (d, *J* = 8.5 Hz, 2H, aromatic), 7.52 (d, *J* = 8.5 Hz, 2H, aromatic), 7.32-7.29 (t, 2H, aromatic), 7.22-7.17 (m, 2H, aromatic), 6.72 (d, *J* = 8.0 Hz, 2H, aromatic), 6.69 (d, *J* = 2.0 Hz, 1H, aromatic), 6.11 (s, 1H, Ar-CH), 3.92, 3.75 (2d, *J* = 16.0 Hz, 2H, SCH_2_); **MS**: m/z (%) 471 (M^+^+2, 5), 469 (M^+^, 15), 368 (9), 247 (18), 233 (73), 231 (100), 196 (16), 168 (31), 139 (38), 93 (87), 70 (72). Anal. Calcd for C_22_H_16_Cl_1_N_3_O_5_S: C, 56.23; H, 3.43; N, 8.94. Found: C, 55.98; H, 3.50; N, 8.92.

#### 4-Chloro-N-(2-(4-methoxyphenyl)-4-oxothiazolidin-3-yl)-2-phenoxybenzamide (5i)

Yield 50 %; m. p 121-123° C**; IR** (KBr) (ν, cm^-1^): 3319 (NH), 1722 (C=O), 1659 (C=O); **^1^H-NMR** (CDCl_3_, 500MHz): δppm 9.01 (s, 1H, NH), 8.13 (d, *J* = 9.0 Hz, 1H, aromatic), 7.38-7.24 (m, 6H, aromatic), 7.14 (d, *J* = 8.5 Hz, 1H, aromatic), 6.77 (d, *J* = 7.5 Hz, 2H, aromatic), 6.72 (d, *J* = 8.5 Hz, 2H, aromatic), 6.64 (s, 1H, aromatic), 5.99 (s, 1H, Ar-CH), 3.91 , 3.68 (2d, *J* = 16.0 Hz, 2H, SCH_2_), 3.66 (s, 3H, CH_3_); **MS**: m/z (%) 457 (M^+^+2,2), 455 (M^+^,6), 233 (51), 231 (100), 208 (96), 196 (21), 168 (33), 151 (28), 134 (60), 93 (55), 77 (78). Anal. Calcd for C_23_H_19_Cl_1_N_2_O_4_S: C, 60.72; H, 4.21; N, 6.16. Found: C, 60.82; H, 4.16; N, 6.09.

#### 4-Chloro-N-(4-oxo-2-p-tolylthiazolidin-3-yl)-2-phenoxybenzamide (5j)

Yield 50 %; m.p. 133-135° C; **IR** (KBr) (ν, cm^-1^): 3326 (NH), 1731 (C=O), 1659 (C=O); **^1^H-NMR** (CDCl_3_, 500MHz): δppm 10.48 (s,1H, NH), 7.43 (d, *J* = 8.3 Hz, 1H, aromatic), 7.38 (t, 2H, aromatic), 7.28-7.18 (m, 4H, aromatic), 7.03 (d, *J* = 7.8 Hz, 2H, aromatic), 6.90 (d, *J* = 7.7 Hz, 2H, aromatic), 6.77 (d, *J* = 1.7 Hz, 1H, aromatic), 5.81 (s, 1H, Ar-CH), 3.87, 3.76 (d, *J* = 15.8 Hz, 2H, SCH_2_), 2.20 (s, 3H, CH_3_); **MS**: m/z (%) 440 (M^+^+2, 9), 438 (M^+^, 27), 247 (35), 233 (78), 231 (100), 192 (59), 168 (56), 155 (33), 139 (62), 118 (34), 93 (82), 77 (38), 57 (15). Anal. Calcd for C_23_H_19_Cl_1_N_2_O_3_S: C, 62.94; H, 4.36; N, 6.38. Found: C, 63.15; H, 4.38; N, 6.29.

### Pharmacology

#### Animals and drugs

For pharmacological tests, we used male NMRI albino mice (weighing 18-22 g; 12 weeks old) obtained from Pasteur Institute (Iran). All animals were kept in the animal house of School of Pharmacy for 7 days in order to adapt to the new place. The mice were kept in standard cages in a typical room in the animal house with controlled conditions designed for experimental animals and have unrestricted access to water and food. Before starting the experiments, mice were also transferred to the testing rooms 60 minutes before the tests to get acclimatized to the experimental condition. We randomly divided the mice into experimental groups and all mice were used only once in the experiments. Ethical Committee of Shahid Beheshti University of Medical Sciences approved all the protocols of the experiments before starting the study. All the tests on animals were done based on NIH publication #85-23. In all protocols we used the least possible number of animals and all ethical advises for work on experimental animals were considered. In this study, diazepam was used as a standard benzodiazepine, and flumazenil was used as antagonist of benzodiazepine receptors. A mixture of DMSO and water (1:10) was used as the solvent for the novel compounds, diazepam, and flumazenil were injected 5 ml/kg i.p.; 30 minutes before the experiments. PTZ, pentobarbital, and midazolam were dissolved in distilled water and injected i.p. (volume of injection was 5 ml/kg). 

#### The anticonvulsant activity 

Two experimental models including PTZ and MES induced convulsions were used to test the anticonvulsant effects of the novel compounds. In PTZ test, different doses of the novel compounds or diazepam were injected and after 30 min, lethal dose of PTZ (100 mg/kg) were injected. We recorded the number of mice protected from PTZ induced convulsion and death for data analysis. In MES model, different doses of the novel compounds or diazepam were injected and after 30 minutes, MES were applied (60 Hz, 37.2 mA and 0.25 s) using ear electrodes. We recorded the number of mice protected from hind limb tonic extension (HLTE) induced by MES for data analysis (Zarghi et al., 2005[24], 2008[22]).

#### Potentiation of pentobarbital sleeping time

For the evaluation of hypnotic effects of the novel compounds and diazepam, we injected different doses of the novel compounds and diazepam (1 mg/kg) and after 30 minutes, injected pentobarbital (65 mg/ kg i.p.) We recorded the duration of loss of righting reflex, which represents the sleeping time. Since pentobarbital may cause hypothermia, animals were located on the top of an electric blanket (set at 37° C) after administration of pentobarbital (Faizi et al., 2015[6]).

#### Passive avoidance test

In order to assess the effect of the novel compounds and midazolam on anterograde memory, previously described step-through passive avoidance test was used. Mice were placed into two compartment, light and dark, apparatus. On the first day (training day) the novel compounds or midazolam were injected and 30 minutes later each animal was located inside the light comportment and let it to freely move. Thirty seconds later the door between two compartments was opened and mouse was able to easily pass the door. Immediately after entering to the dark compartment, an unpleasant electrical shock (0.5 mA, 2 seconds) was delivered to the mouse from bottom of the compartment. On the second day (testing day), animal was located in the light compartment and the delay time for each mouse to enter the dark compartment was recorded (Faizi et al., 2015[6]).

#### Neurotoxicity evaluation

The neurotoxicity of the compound **5i** was measured in mice by the rotarod test. The mice were trained to stay on a rotarod of diameter 1 inch that rotates at 6 rpm and were given i.p. of the **5i**. If in each trial mouse was not able to stay at least 1 minute on the rotarod, it was considered as neurotoxicity-induced deficit in coordination or balance (Sałat et al., 2013[17]).

#### Data analysis

ED_50_ of the anticonvulsant effects of the all tested compounds were calculated by probit-regression analysis using SPSS software (Chicago, IL; 1 version 13). To compare the ED_50s_, Fisher's exact probability test was used. Results of PTZ and MES experiments were reported as mean with 95 % confidence intervals. Results of the other experiments were reported as mean ± SEM and one-way analysis of variance (ANOVA) with Tukey's HSD post hoc test were used to analyse the results. In all tests, if *P*-value was less than 0.05, we concluded that a significant difference existed.

## Results and Discussion

### Prediction of the BBB permeably of compounds

Anticonvulsant agents similar to other CNS active compounds should be able to pass through blood-brain barrier (BBB). So the *in silico* prediction of the BBB permeably of the designed compounds was done by the BBB prediction server which is a part of AlzPlatform (Liu et al., 2014[12]), using support vector machine (SVM_Molptint 2D) algorithm (http://www.cbligand.org/BBB). The ability or inability of a compound to penetrate blood-brain barrier (BBB+/BBB-) depends on the BBB score in the selected algorithm. When it is more than zero, compound could be consider as BBB+ and the best score is in the range of 0.2-0.4. The scores of the designed compounds were in the range of 0.139-0.681, so it was deduced that all of the novel compounds are BBB+ and the density of compounds **5a**, **5d-5f**, **5i-5j** in the CNS could be more than other compounds.

### Physicochemical properties

The physicochemical properties and the Lipinski's rule of five parameters for prediction of oral bioavailability were determined by molinspiration online program (www. molinspiration.com) and also the clogp was achieved by Osiris Property Explorer (OPE) (http://www.organic-chemistry.org/prog/peo/) (Table 1(Tab. 1)) (Lipinski et al., 2012[11]). According to the Lipinski's rule for oral absorption of compounds, their molecular weight should be lower than 500, the number of hydrogen bond donor and acceptor should be ≤ 5 and ≤ 10 respectively, the topological polar surface area should be ≤ 140 and number of rotatable bonds ≤ 10 and two violations of this rule will result to the poor oral absorption. As shown in Table 1(Tab. 1), all compounds except compound **5c** can be absorbed orally (Table 1(Tab. 1)). 

### Chemistry

**Figure 2:**
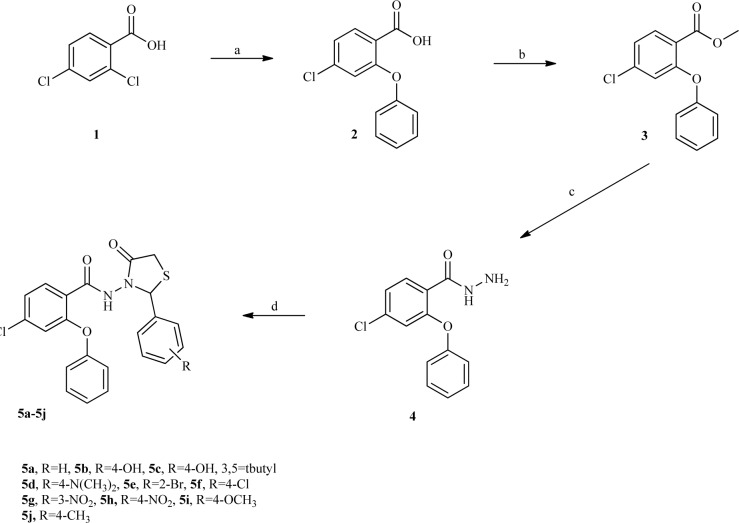
Synthesis of the novel thiazolidinone compounds 5a-5j. (a) phenol, NaH 60 %, Cu, Dry DMF, reflux, 20h, 61 %; (b) H_2_SO_4_, MeOH, reflux, 24h, 87 %; (c) NH_2_NH_2_.H_2_O, EtOH, rt, 24h, 93 %; (d) 1) appropriate benzaldehyde, HCl 37 %, absolute EtOH, rt, 2-20h 2) thioglycolic acid, ZnCl_2_, dry toluene, reflux, 20 h, 50-64 %

The designed compounds were synthesized according to the Figure 2(Fig. 2), 4-Chloro-2-phenoxybenzoic acid **1** was synthesized through a nucleophilic aromatic substitution reaction of 2,4-dichlorobenzoic acid and phenol. Following esterification of **1**, 4-chloro-2-phenoxybenzohydrazide **3, **was prepared in satisfactory yield by the reaction of ester **2** with hydrazine hydrate at room temperature (Rezaee et al., 2014[14][15]). Treatment of hydrazide **3** with corresponding benzaldehyde followed by reaction with thioglycolic acid afforded final products (**5a-5j**) (Shingalapur et al., 2010[18]; Almasirad et al., 2006[3]). The final compounds were characterized through ^1^H-NMR, FT-IR, Mass spectra and elemental analysis.

### Pharmacology

#### The anticonvulsant activity

PTZ induced lethal convulsion test and MES test were used for evaluation of anticonvulsant effects of the compounds. Results shown in Table 2(Tab. 2) indicate that, compounds **5a**, **5b**, **5c**, **5d**, **5e**, **5f**, **5g**, **5h **and **5j **were inactive in PTZ model. Compound **5i** was the only compound, which showed significant anticonvulsant effect in PTZ model. Compounds** 5c**, **5d**, **5g** and **5j** had no significant anticonvulsant effect in the MES model but other compounds, including **5i,** showed significant activity in this model. According to the lower ED_50_, it could be concluded that **5i **was the most potent compound between the novel thiazolidinone derivatives in the two models. In both MES and PTZ tests, flumazenil was able to significantly reduce the effect of the compounds with anticonvulsant activity.

#### Potentiation of pentobarbital sleeping time

**Figure 3:**
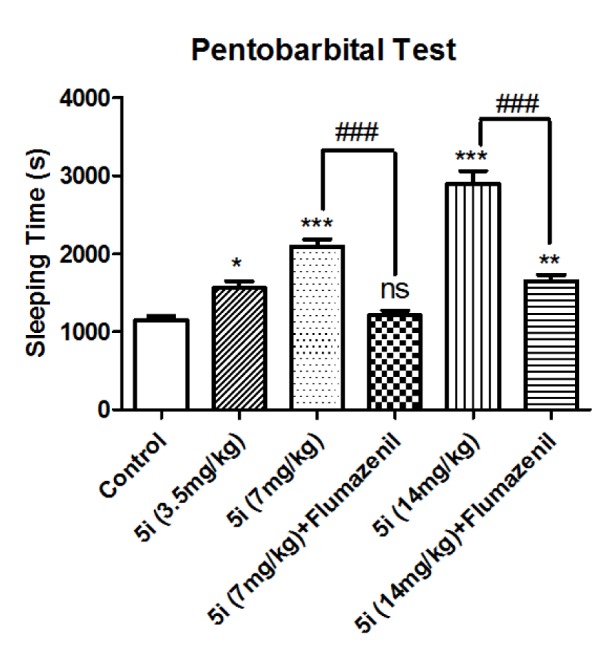
Effect of compound 5i on potentiation of pentobarbital sleeping time: the time period of loss of righting reflex, which is conidered as sleeping time is presented. Results are shown as mean±SEM. In all groups n=10. ns represents not significant, * represents* p*<0.05, ** represents *p*<0.01, and *** represents *p*<0.001 compared to the control group. ### represents p<0.001comparing two indicated groups

Hypnotic effect of compound **5i** was evaluated by increasing pentobarbital sleeping time. According to Figure 3(Fig. 3), the sleeping time was increased by the injection of compound **5i** and the effect was dose-dependent. However, the pharmacological effects of compound **5i** were blocked after injection of flumazenil, which indicates that BZD receptors are responsible for these effects.

#### Passive avoidance test

**Figure 4:**
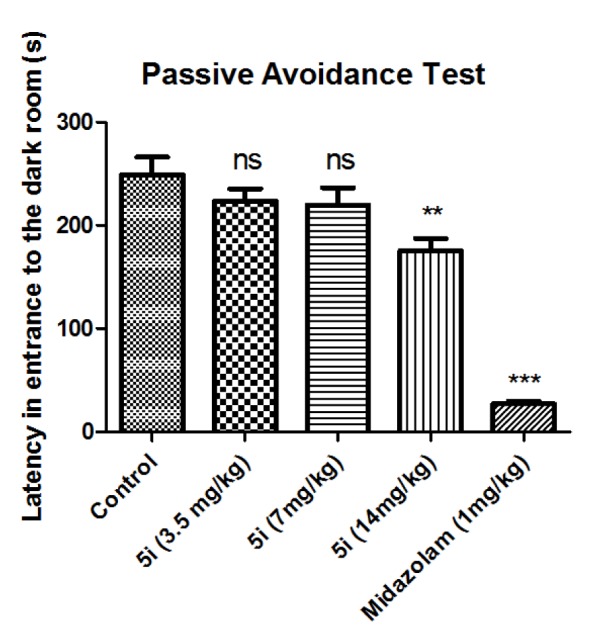
Effect of compound 5i on memory retrival in step-through passive avoidance experiment: Results of the latencies to enter the dark compartment on the second day are presented. Results are shown as mean±SEM. In all groups n = 10. ns represents not significant, ** represents *p *< 0.01, and *** represents *p *< 0.001 compared to the control group

The passive avoidance test was used to evaluate the effects of compound **5i** on anterograde memory. As shown in Figure 4(Fig. 4), compound **5i** in doses of 3.5 and 7 mg/kg had no destructive effect on anterograde memory comparing to the control group, but in dose of 14 mg/kg the anterograde amnesia was induced by this compound. 

#### In silico toxicity evaluation

The *in silico* toxicity risk assessment was performed by means of OPE and except compound **5e** which showed high risk of tumorigenic effect all of them can be considered as non-mutagenic, non-tumorigenic, non-irritant without any reproductive effects.

#### Neurotoxicity evaluation

**Figure 5:**
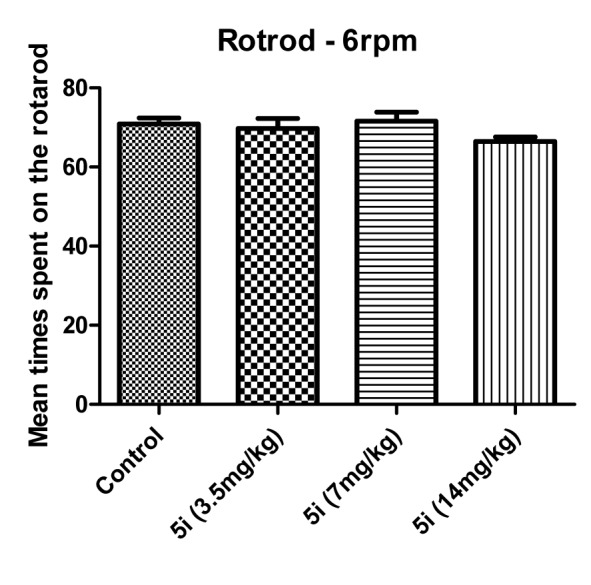
Influence of the test compounds 5i (at 3.5, 7 and 14 mg/kg) on motor coordination of experimental animals measured in the rotarod test revolving at 6 rpm. Results are shown as mean time spent on the rotating rod.

According to Figure 5(Fig. 5), compound **5i** exhibited no neurotoxicity effect in doses of 3.5, 7 and 14 mg/kg. According to the BBB+ scores of compounds **5c**, **5g** and **5h**, their inactivity could be due to the low density in CNS. As shown in Table 2(Tab. 2) compound **5b** and **5h** are active in the MES model but their potency is less than **5a**, **5e** and **5i.** This may be due to their lower concentrations in the CNS, not less binding affinity to the receptor. 

## Conclusion

In summary, the new 4-thiazolidinone derivatives as novel BZD agonists were synthesized and their biological effects were investigated. Some of the novel synthesized compounds showed antiepileptic effects in PTZ and MES tests. Compound **5i** had the best anticonvulsant activity among the novel 4-thiazolidinone compounds and revealed considerable hypnotic effect. However, it did not change the antrograde memory and did not show neurotoxicity. Cosidering the fact that the most clinically used benzodiazepins have negative effect on memory, the novel 4-thiazolidinone derivatives (specially compound **5i**) can be used as leading compound and related derivatives can be designed and synthesized as novel ligands for benzodiazepine receptors which has minimum effect on memory. 

## Acknowledgements

This work was supported by a grant (No.1271) from the Research Council of Shahid Beheshti University of Medical Sciences.

## Conflict of interest

The authors confirm that this article content has no conflict of interest.

## Figures and Tables

**Table 1:**
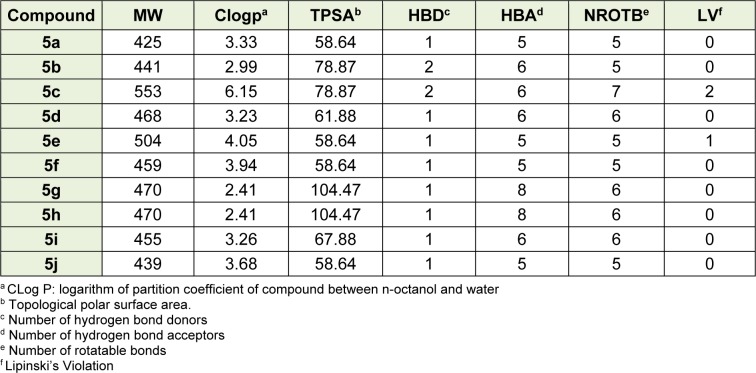
Pharmacokinetic parameters important for good oral bioavailability of the synthesized compounds 5a-5j

**Table 2:**
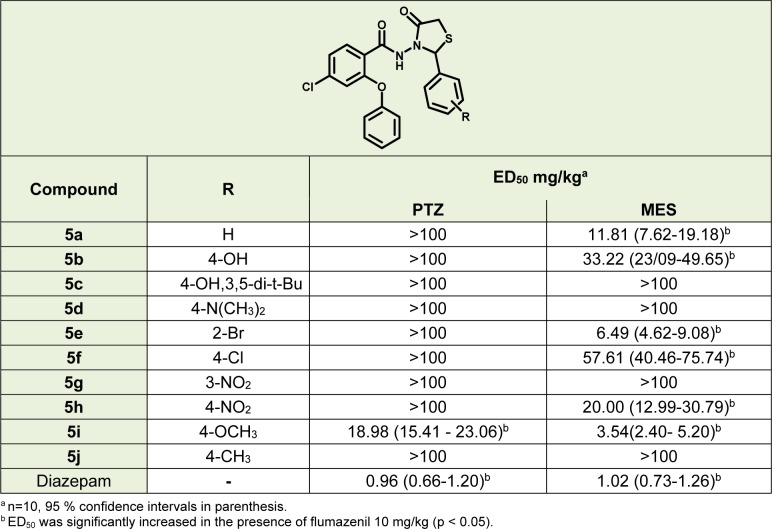
ED_50_ of the novel thiazolidinone compounds and diazepam in PTZ and MES tests in mice
